# Synthesis of Ball-Like Ag Nanorod Aggregates for Surface-Enhanced Raman Scattering and Catalytic Reduction

**DOI:** 10.3390/nano6060099

**Published:** 2016-05-27

**Authors:** Wenjing Zhang, Yin Cai, Rui Qian, Bo Zhao, Peizhi Zhu

**Affiliations:** 1 School of Chemistry and Chemical Engineering, Yangzhou University, Jiangsu 225002, China; 15262236582@163.com (W.J.Z.); yincai1992@sina.com (Y.C.); ruiqian2016@sina.com (R.Q.); 2 Jiangsu Collaborative Innovation Center of Biomedical Functional Materials and Jiangsu Key Laboratory of Biofunctional Materials, School of Chemistry and Materials Science, Nanjing Normal University, Nanjing 210023, China; zhaobo@njnu.edu.cn

**Keywords:** Ag nanorod aggregates, surface-enhanced Raman scattering, Rhodamine 6G, doxorubicin, PVP, catalytic reduction, 4-nitrophenol

## Abstract

In this work, ball-like Ag nanorod aggregates have been synthesized via a simple seed-mediated method. These Ag mesostructures were characterized by scanning electron microscope (SEM), transmission electron microscopy (TEM), ultraviolet-visible spectroscopy (UV-Vis), and X-ray diffraction (XRD). Adding a certain amount of polyvinyl pyrrolidone (PVP) can prolong its coagulation time. These Ag nanorod aggregates exhibit effective SERS effect, evaluated by Rhodamine 6G (R6G) and doxorubicin (DOX) as probe molecules. The limit of detection (LOD) for R6G and DOX are as low as 5 × 10^−9^ M and 5 × 10^−6^ M, respectively. Moreover, these Ag nanorod aggregates were found to be potential catalysts for the reduction of 4-nitrophenol (4-NP) in the presence of NaBH_4_.

## 1. Introduction

In recent years, silver nanoparticles (AgNPs) have seen broad application in areas such as catalysis [1], biomedicine [2], antimicrobial agent [3] and SERS [4,5]. As a powerful molecular fingerprinting technique, surface-enhanced Raman scattering (SERS) is a sensitive technique for trace detection [6,7,8,9,10]. Noble metal nanoparticles such as Ag and Au particles have been extensively explored due to their high SERS-active properties [11,12,13,14,15,16,17]. It is well-established that SERS activities are size and shape dependent [18,19]. Ag nanoparticles with complex topography have more hot-spots on surface to amplify Raman scattering of probe molecules [20,21].

Silver nanoparticles have also gained much attention for their application as a sustainable catalyst for organic transformations owing to their unique electronic properties and high surface area to volume ratio [22]. Particularly, silver nanoparticles show highly efficient catalytic activity in oxidation of methanol and ethylene [23,24], as well as reduction of nitric oxides (NO*_x_*) [25]. Yang *et al*. [26] proposed that the flower-like Ag microcrystal exhibited high catalytic activity for 4-nitrophenol reduction due to their high surface area and the local electromagnetic field intensity enhancement.

Many researchers have studied numerous Ag complex structures as highly sensitive SERS substrates and catalysts [27,28,29,30,31]. Various methods such as chemical reduction [32,33], template process [34], and galvanic replacement [35] have been used to synthesize functional Ag nanoparticles. Using a double-reductant approach, seed-mediated method has been explored to prepare Ag nanocubes [36,37], nanowires [38], nanopolyhedron [39], and gold-Ag nanoparticles [40]. However, complex Ag structures possess larger surface area than single Ag nanoparticles and easily aggregate. One main method to enhance the stabilization of Ag nanoparticles is to use polymers or surfactants to modify the surface of Ag particles to prevent particles from aggregating. Being a nonionic polymer compounds, polyvinyl pyrrolidone (PVP) is often used as the capping agent to control the size and shape of the colloidal nanoparticles including Ag-NPs, Au-NPs, and Pt-NPs during the particle formation [41,42,43]. PVP has also been reported to be a reducing agent in the preparation for the hydroxyl end-group of the PVP polymer chain [44].

In this study, we synthesized ball-like Ag nanorod aggregates via a simple seed-mediated method without any surfactant and polymeric compound as a capping reagent in reduction. PVP was added in the last step and was used only as a stabilizer. The present approach is simple, economic and green. The SERS properties of these Ag nanorod aggregates were examined by using Rhodamine 6G (R6G) and doxorubicin (DOX) as probe molecules. In addition, its catalytic performance for the reduction of 4-nitrophenol (4-NP) in the presence of NaBH_4_ was also examined.

## 2. Results and Discussion

### 2.1. Phase Characterization

**Figure 1:**
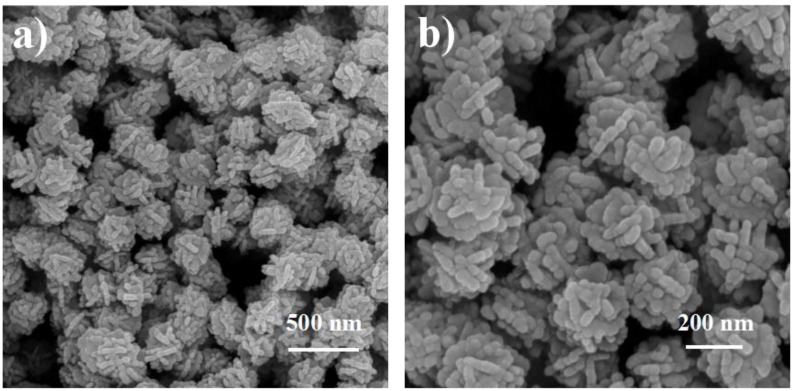
Scanning electron microscope (SEM) images of the ball-like Ag nanorod aggregates.

**Figure 2:**
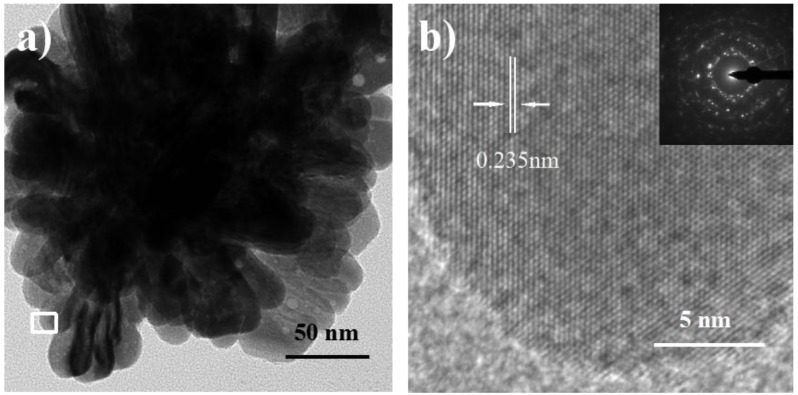
Transmission electron microscopy (TEM) (**a**) and high resolution TEM (HRTEM) (**b**) images of the ball-like Ag nanorod aggregates. Inset in (**b**) is the selected area electron diffraction (SEAD) pattern of Ag nanorod aggregates.

Ball-like Ag nanorod aggregates were synthesized via a seed-mediated method involving two reaction steps without using any surfactant and polymeric compound as a capping reagent in reduction. After adding 25 mL of 20 mM AgNO_3_, aggregates comprising dozens of Ag nanorods with a mean size of about 180 nm were formed (Figure 1). It is observed that these nanorods exhibit lengths of ~50 nm and diameters of ~20 nm (Figure 1b). The polycrystalline SEAD (selected area electron diffraction) pattern of in Figure 2b confirms the diverse orientations of these nanorods in aggregates. Figure 2b shows the HRTEM image of clear lattice fringes with the spacing of 0.235 nm, which corresponds to the (111) lattice planes of the fcc-Ag [45].

### 2.2. UV-Vis Studies of Ag Nanorod Aggregates

**Figure 3:**
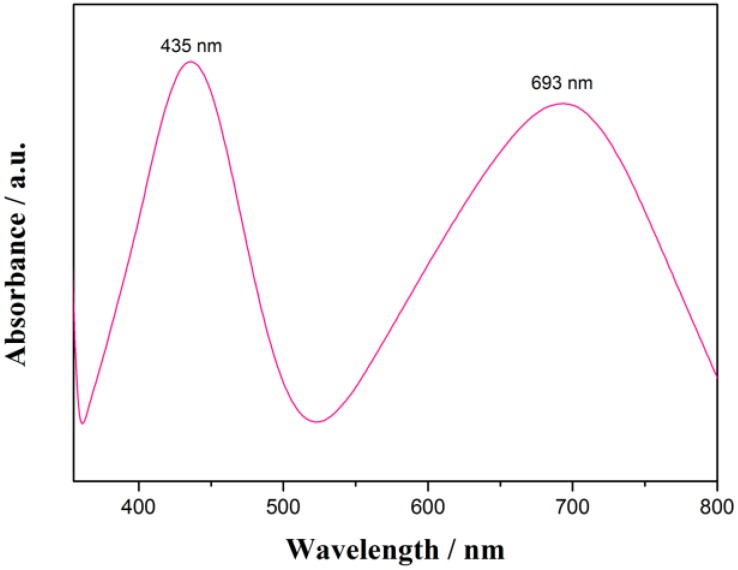
Ultraviolet-visible spectroscopy (UV-Vis) spectrum of the ball-like Ag nanorod aggregates.

It is well-known that the size and shape of metal nanoparticles could affect their optical properties such as surface plasmon resonance (SPR) property [46,47]. For instance, AgNPs with complex structures usually exhibit more than one peak [48,49], whereas spherical particles show only one size-dependent SPR peak [50]. As shown in Figure 3, the spectrum of Ag nanorod aggregates in aqueous solution displays two SPR bands that might indicate the information of nonspherical AgNPs. The lower wavelength band (435 nm) could be attributed to the out-of plane dipole resonance while the 693 nm peak (the high wavelength band) is in-plane dipole resonance [48]. In each nanorod-aggregate, the conduction electrons near each nanorod surface become delocalized and are shared amongst neighboring nanorods, which shifts the surface plasmon resonance to lower energies, moves the absorption peak to longer wavelengths and broadens the absorption spectrum.

### 2.3. XRD Studies of Ag Nanorod Aggregates

**Figure 4:**
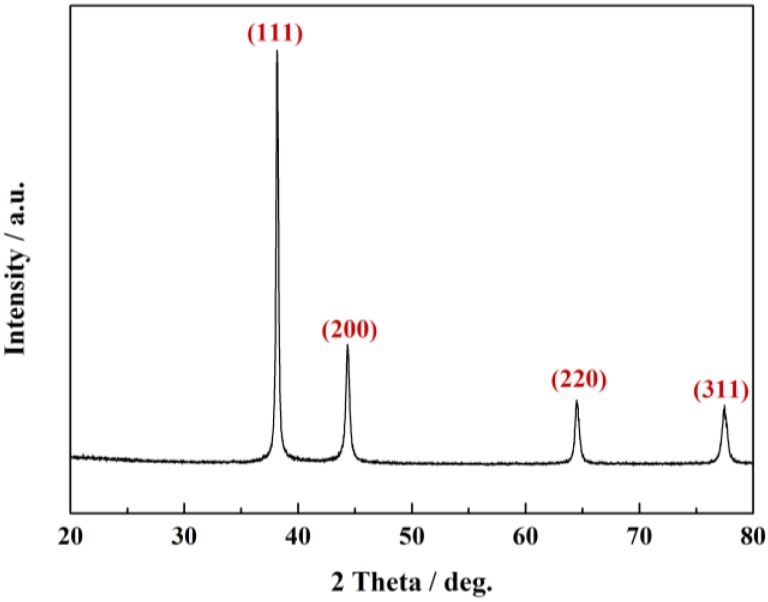
X-ray diffraction (XRD) pattern of the ball-like Ag nanorod aggregates.

The structure of prepared ball-like Ag nanorod aggregates has been studied by X-ray diffraction (XRD) analysis. A typical XRD pattern of the particles was shown in Figure 4. The sharp peaks in XRD pattern prove the high crystallinity of Ag nanorod aggregates. The four diffraction peaks observed 38.17°, 44.28°, 69.45°, and 77.49° are corresponding to (111), (200), (220), and (311) Bragg’s reflections of the face-centered cubic structure of Ag, respectively (JCPDS ICDD 04–0783) [51]. There is no peak of other impurities being found from the pattern, which indicates pure Ag crystals were obtained under the present method.

### 2.4. Formation Mechanism of Ag Nanorod Aggregates

**Figure 5:**
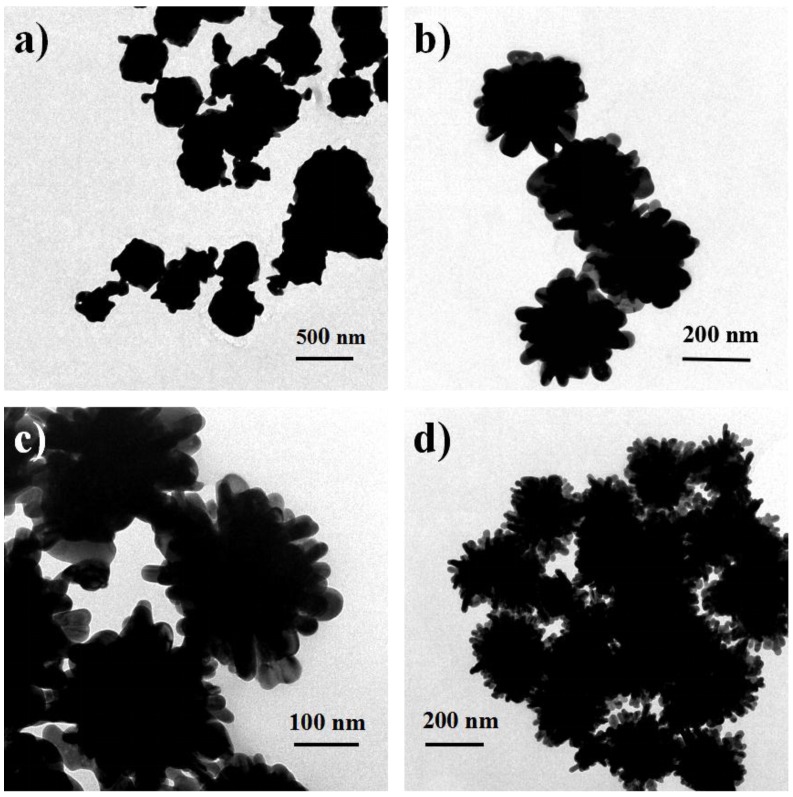
TEM images of the ball-like Ag nanorod aggregates under different concentrations of AgNO_3_: (**a**) 5 mM; (**b**) 10 mM; (**c**) 15 mM; (**d**) 20 mM.

The morphology of Ag nanoparticles influences their applications. In the synthesizing process of metal nanoparticles, the morphology of nanoparticle can be controlled by adjusting the reaction time, the concentration of the precursor and the reactants, and so on [30]. In our synthesis process, the reaction was almost instantaneous. Hence, the reaction rate is not main consideration. Herein, the added Ag seeds serve as the nucleation sites for the growth of the Ag nanorod aggregates. Since the ascorbic acid used as the reducing agent in second step is excessive, the anisotropic growth process could be dominated by the amount of Ag^+^ ions, namely the concentration of AgNO_3_. As shown in Figure 5, when the concentration of AgNO_3_ varied from 5 mM to 20 mM, Ag nanorod aggregates show similar diameters but different morphologies. At low concentration of AgNO_3_, a great quantity of near-spherical particles is produced. At higher concentration of AgNO_3_, ball-like Ag nanorod aggregates are formed, indicating that the concentration of AgNO_3_ is key factor for forming ball-like Ag nanorod aggregates [20].

### 2.5. Stability Analysis of Ag Nanorod Aggregates

**Figure 6:**
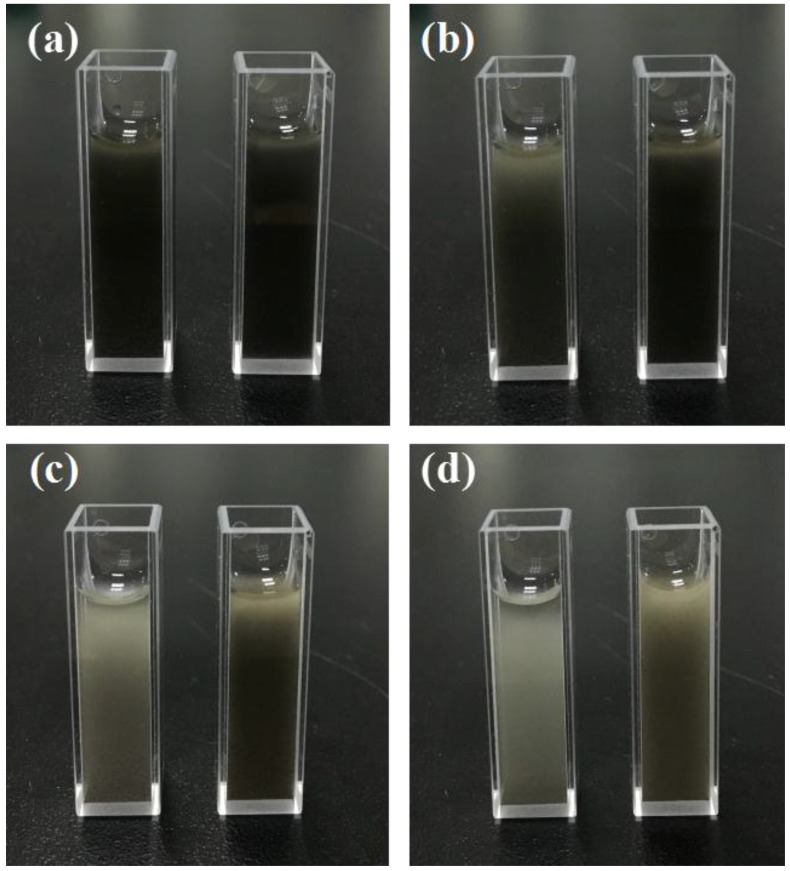
The coagulation condition of Ag nanorod aggregates without adding polyvinyl pyrrolidone (PVP) (left) and with adding PVP (right): (**a**) 0 min; (**b**) 10 min; (**c**) 30 min; (**d**) 80 min.

The aggregation of ball-like Ag nanorod aggregates is a concern for application that may take more time to handle with. To solve this problem, 0.005% wt % of PVP has been used in an effort to stabilize large size Ag particles in aqueous solution. Figure 6a shows the freshly obtained Ag nanorod aggregates without adding PVP (left) and with adding PVP (right). After 10 min, as shown in Figure 6b, Ag nanorod aggregates without adding PVP began to coagulate, while Ag nanorod aggregates with adding PVP remained stable due to the interaction between the particles and carbonyl groups on polymer chains of PVP. After 30 min (Figure 6c), Ag nanorod aggregates without adding PVP precipitated to the bottom of the bottle, and Ag nanorod aggregates with PVP begin to precipitate. However, as is shown in Figure 6d, Ag nanorod aggregates with PVP remained relatively stable even after 80 min compared with Ag nanorod aggregates without PVP. Therefore, PVP can serve as an effective stabilizer.

### 2.6. SERS Performances of Ag Nanorod Aggregates

**Figure 7:**
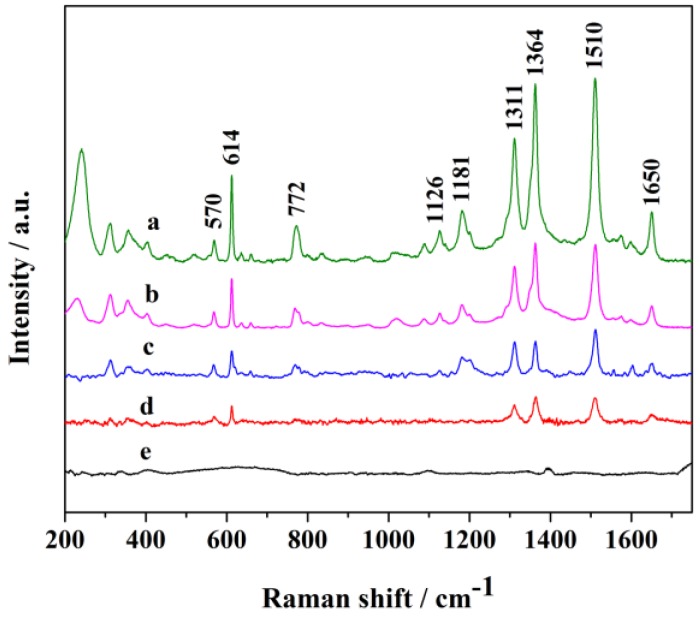
Raman spectra of Rhodamine 6G (R6G) at different concentrations absorbed on Ag nanorod aggregates. Spectra represent the concentrations of R6G being (**a**) 5 × 10^−6^; (**b**) 5 × 10^−7^; (**c**) 5 × 10^−8^; (**d**) 5 × 10^−9^; (**e**) 5 × 10^−10^ M, respectively.

It is critical to determine the practical limit of detection (LOD) of probe molecules in SERS applications. Accordingly, the practical LOD of R6G absorbed on ball-like Ag nanorod aggregates coated with PVP in this work was discussed. Ag nanorod aggregates formed by self-assembled nanorods. The gaps between nanorods generate active sites or hot-spots to amplify Raman scattering of probe molecules. The Raman spectra of R6G with different concentrations absorbed on AgNPs were displayed in Figure 7. All peaks of R6G in spectra agree well with previous report [52]. PVP does not produce Raman signal at such a low concentration. The peaks at 1364, 1510 and 1650 cm^−1^ are attributed to the aromatic C–C stretching modes of R6G molecules, while the peak at 772 cm^−1^ is assigned to the C–H out-of-plane bend mode. As shown by spectrum d (5 × 10^−9^ M), the characteristic bands of R6G at 570, 614, 1311, 1364, 1510, 1650 cm^−1^ can be still clearly detected. Therefore, the LOD for R6G absorbed on Ag nanorod aggregates was identified as 5 ×10^−9^ M. It is difficult to calculate the enhancement factor of the R6G molecule under available experimental conditions. Hence, we calculate the relative enhancement factor for peak at 1510 cm^−1^ by calculating the Raman intensity ratios between 5 × 10^−6^ M and 5 × 10^−10^ M. The relative enhancement factor is calculated to be 3.3 × 10^3^, indicating that the flower-like nanorod aggregates could serve as effective SERS substrate.

**Figure 8:**
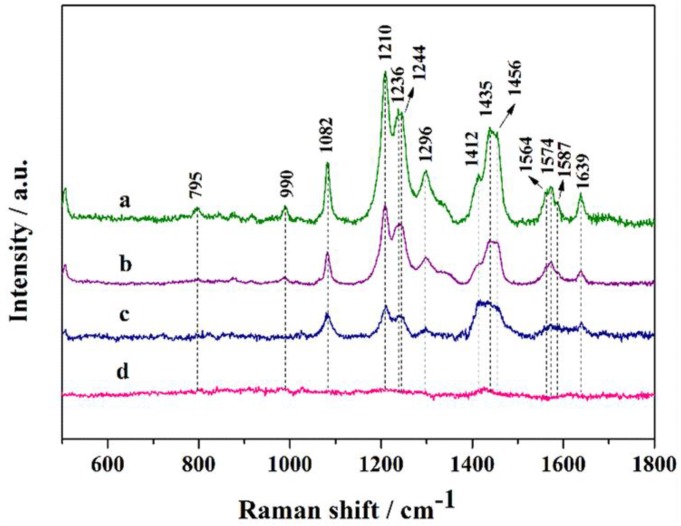
Raman spectra of DOX at different concentrations absorbed on Ag nanorod aggregates. Spectra represent the concentrations of DOX being (**a**) 5 × 10^−4^; (**b**) 5 × 10^−5^; (**c**) 5 × 10^−6^; (**d**) 5 × 10^−7^ M, respectively.

Doxorubicin is commonly used as chemotherapy drug for patients with advanced cancers. SERS has been used as a powerful tool to study DOX complexes with DNA [53] and its affinity for ferric ions. In this study, we also used DOX as probe molecule to test SERS effect of Ag nanorod aggregates. Figure 8 shows the SERS spectra of DOX at different concentrations. The band at 1639 cm^−1^ is assigned to the stretching mode of carbonyl groups [54]. The band at 1296 cm^−1^ is from C–O stretching and the two strong bands at 1244 and 1210 cm^−1^ can be assigned to in-plane bending motions from C–O. The weak bands at 1082 and 795 cm^−1^ are assigned to skeletal deformations, while 990 cm^−1^ is owing to ring breath modes. When the concentration of DOX reduces to 5 × 10^−6^ M, bands at 1082, 1210, 1244, 1412, 1435, 1456 and 1639 cm^−1^ can still be clearly detected. Thus, the LOD for DOX absorbed on Ag nanorod aggregates was identified as 5 × 10^−6^ M. Hence, the flower-like Ag nanorod aggregates could serve as SERS substrate for trace analysis for small drug molecules.

### 2.7. Catalytic Reduction of 4-Nitrophenol

**Figure 9:**
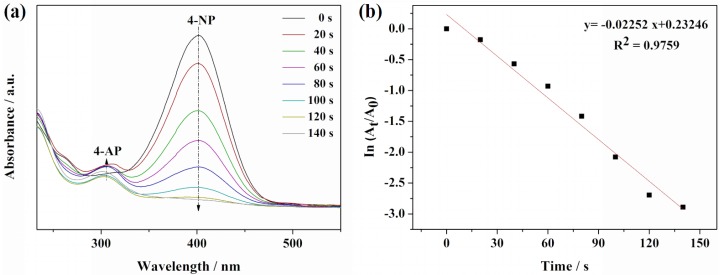
UV-Vis absorption spectra: (**a**) reduction of 4-NP by NaBH_4_ using Ag nanorod aggregates as catalyst; (**b**) The plot of ln(*A*_t_/*A*_0_) against the reaction time for pseudo-first-order reduction kinetics of 4-NP in the presence of ball-like Ag nanorod aggregates.

The reduction of 4-nitrophenol to 4*-*aminophenol (4-AP) by NaBH_4_ was taken as a model reaction to examine the catalytic activity of the ball-like Ag nanorod aggregates. It is well known that the absorption peak of 4-NP with light yellow color is around 317 nm [55]. After the addition of freshly prepared NaBH_4_ solution, the light yellow turned to intense yellow, which indicates the formation of 4-nitrophenolate ion and the pH change from acid to basic by adding NaBH_4_. The catalytic process of this reaction was monitored by UV-Vis spectroscopy, Figure 9a shows the UV-Vis spectra of 4-NP reduction in the presence of NaBH_4_ and 0.2 mL of Ag nanorod aggregates. As shown in Figure 8a, absorption band at 400 nm is characteristic peak of the 4-nitrophenolate in the presence of only NaBH_4_. However, after adding 0.2 mL of ball-like Ag nanorod aggregates as a catalyst, a new band at around 300 nm emerged, indicating reduction of 4-NP to 4-AP by NaBH_4_ (Figure 9a). The intensity of the absorption peak at 400 nm gradually decreased with time, while absorption peak at 300 nm increased simultaneously (Figure 9a). Until the intensities of two peaks no longer changed, the reduction finished. The extinction of solution at 400 nm as the function of time was measured to monitor the kinetic process of the reduction. The rate constant (*K*) was contingent upon reduction time and the linear plot of ln (*A*_t_/*A*_0_), following pseudo-first-order kinetics (Figure 9b). The constant was calculated to be 0.02252 s^−1^, proving that the ball-like Ag aggregates is effective catalyst for the reduction of 4-NP. Usually, the catalytic activity is influenced by the surface area and roughness of the catalyst. Obviously, the good catalytic performance of the ball-like Ag nanorod aggregates could be attributed to their high surface area to volume ratio.

## 3. Materials and methods

### 3.1. Materials

Silver nitrate (AgNO_3_, 99.8%), tri-sodium citrate dihydrate (C_6_H_5_Na_3_O_7_·2H_2_O, 99%), L-ascorbic acid (Vitamin C, 99.7%), polyvinyl pyrrolidone (PVP, MW ≈ 45,000 daltons), Rhodamine 6G, Doxorubicin hydrochloride (DOX·HCl) was obtained from Fortuneibo-tech Co., Ltd (Shanghai, China). 4-nitrophenol (4-NP), sodium borohydride (NaBH_4_, 96%) were purchased from Sinopharm Chemical Reagent Co. Ltd (Shanghai, China). All the chemicals were of analytical reagent grade and were used without further purification. All of the solutions were freshly prepared using deionized double-distilled water from a Milli-Q water purification system (Millipore Corporation, Billerica, MA, USA).

### 3.2. Preparation of Ag Aggregates

In a typical experiment, 200 mL of 2.5 mM aqueous solution of AgNO_3_ were brought to boiling. Then, an aqueous solution of 100 mM sodium citrate (10 mL) was added dropwise in to the boiled AgNO_3_ solution at a rate of 30 drops per min. After retained boiling for 10 min, the colorless solution turned to yellowish and turbid colloid characteristic of the seeds formation. In order to get Ag nanorod aggregates, 5 mL of Ag seed was diluted into 35 mL freshly prepared L-ascorbic acid (Vitamim C) solution (5 mM). Subsequently, 25 mL of AgNO_3_ at a certain concentration was directly added. A generation of dark grey suspension indicated the formation of Ag nanorod aggregates. All the reactions were kept in the dark to avoid any photoreaction.

Later, 0.003 g of PVP was added to generated Ag suspension and the above mixture was performed by 5 min ultrasonic treatment. The coagulation condition of Ag particles was recorded by taking photos every 10 min in a while.

### 3.3. Characterization Techniques

Scanning electron microscopy (SEM, S-4800Ⅱ, Hitachi, Tokyo, Japan) and transmission electron microscopy (TEM, Philips Tecnai 12, Amsterdam, the Netherlands) was used to observe the morphologies and particle sizes of Ag nanorod aggregates. Transmission electron microscopy was performed by fixation on a 200-mesh carbon-coated copper grid. Ag nanorod aggregates were dropped onto clear glass slide and the glass slide was dried at room temperature. Then, the absorbance spectrum of Ag nanorod aggregates was measured by a UV-Vis spectroscopy (Varian Cary 500, Palo Alto, CA, USA) in the range of 350 to 800 nm. X-ray diffraction (XRD, D8 ADVANCE, Bruker, Karlsruhe, Germany) with graphite monochromatized Cu Kα radiation operating at 40 kV and 40 mA at room temperature in the range 2θ (20° ≤ 2θ ≤ 80°) was utilized to determine the crystalline structure of the samples.

### 3.4. SERS Performance of R6G and DOX on Ag Nanorod Aggregates

To determine the LOD for R6G and DOX, a series of concentrations of R6G and DOX in water were detected using SERS Ag nanorod aggregates coated with PVP. SERS spectra were recorded with a Confocal Raman spectrometer (DXR, GX-PT-2412, Thermo, Waltham, MA, USA) with 780 nm line of a He-Ne laser as excitation wavelength. The laser power at the samples was 24 mW and the data acquisition time was 60 s. R6G and DOX was used as probe molecules. 200 µL of R6G at concentration of 5 × 10^−6^, 5 × 10^−7^, 5 × 10^−8^, 5 × 10^−9^, 5 × 10^−10^ M were mixed with 200 µL of Ag nanorod aggregates suspension at concentration of 8.3 mM. 200 µL of DOX at concentration of 5 × 10^−4^, 5 × 10^−5^, 5 × 10^−6^, 5 × 10^−7^ M were mixed with 200 µL of Ag nanorod aggregates suspension at concentration of 8.3 mM. The spectra were obtained in solution-phase after mixing for an hour to make sure that dye molecules could absorb on the surface of AgNPs sufficiently at room temperature.

### 3.5. Catalytic Reduction

In a typical run for the reduction of 4-NP by NaBH_4_, 0.05 mL of fresh solution of 4-NP (1 mM) was introduced into 2 mL of NaBH_4_ (0.1 M) solution. Then, 0.2 mL of Ag nanorod aggregates (1 mM) was added to the above mixed solution. Then, with the addition of 2.75 mL of ultrapure water, the total volume of the reaction system was 5.00 mL. Set a blank group, the reaction system is same as the above system except that the Ag nanorod aggregates solution was replaced by ultrapure water. After the addition of 0.2 mL of Ag nanorod aggregates catalyst, scanning 600–250 nm band immediately in order to monitor the spectra of the 4-NP reduction in the presence of NaBH_4_ and Ag nanorod aggregates solution by using UV-visible spectrophotometric monitoring instrument.

## 4. Conclusions

In summary, ball-like Ag nanorod aggregates with a mean size of 180 nm were synthesized by a seed-mediated approach, which is simple, economic and green. The polyvinyl pyrrolidone (PVP) was used to enhance the stability of obtained Ag nanorod aggregates in aqueous solution for SERS and catalytic experiment. Ag nanorod aggregates exhibit effective and reproducible SERS effect, evaluated by R6G as probe molecules. The limit of detection (LOD) for R6G and DOX are as low as 5 × 10^−9^ M and 5 × 10^−6^ M, respectively, which shows promising application for trace detection of small molecules. These Ag nanorod aggregates possess large surface area and porous surface morphology and could serve as a potential catalyst for the reduction of 4-NP in the presence of NaBH_4_.
